# Understanding the Associations of Prenatal Androgen Exposure on Sleep Physiology, Circadian Proteins, Anthropometric Parameters, Hormonal Factors, Quality of Life, and Sex Among Healthy Young Adults: Protocol for an International, Multicenter Study

**DOI:** 10.2196/29199

**Published:** 2021-10-06

**Authors:** Wojciech Kuczyński, Erik Wibowo, Tetsuro Hoshino, Aleksandra Kudrycka, Aleksandra Małolepsza, Urszula Karwowska, Milena Pruszkowska, Jakub Wasiak, Aleksandra Kuczyńska, Jakub Spałka, Paulina Pruszkowska-Przybylska, Łukasz Mokros, Adam Białas, Piotr Białasiewicz, Ryujiro Sasanabe, Mark Blagrove, John Manning

**Affiliations:** 1 Department of Sleep Medicine and Metabolic Disorders Medical University of Lodz Lodz Poland; 2 Department of Anatomy School of Biomedical Sciences University of Otago Dunedin New Zealand; 3 Department of Sleep Medicine and Sleep Disorder Center Aichi Medical University Aichi Japan; 4 Department of Microbiology and Laboratory Medical Immunology Medical University of Lodz Lodz Poland; 5 Department of Anthropology Faculty of Biology and Environmental Protection University of Lodz Lodz Poland; 6 Department of Clinical Pharmacology Medical University of Lodz Lodz Poland; 7 Department of Pathobiology of Respiratory Diseases Medical University of Lodz Lodz Poland; 8 Department of Clinical and Experimental Physiology Medical University of Lodz Lodz Poland; 9 Department of Psychology Swansea University Swansea United Kingdom; 10 Applied Sports, Technology, Exercise, and Medicine Research Centre Swansea University Swansea United Kingdom

**Keywords:** digit ratio, sleep, sex hormones, testosterone, estrogen, circadian proteins, circadian rhythm, chronotype, miRNA

## Abstract

**Background:**

The ratio of the second finger length to the fourth finger length (2D:4D ratio) is considered to be negatively correlated with prenatal androgen exposure (PAE) and positively correlated with prenatal estrogen. Coincidentally, various brain regions are sensitive to PAE, and their functions in adults may be influenced by the prenatal actions of sex hormones.

**Objective:**

This study aims to assess the relationship between PAE (indicated by the 2D:4D ratio) and various physiological (sex hormone levels and sleep-wake parameters), psychological (mental health), and sexual parameters in healthy young adults.

**Methods:**

This study consists of two phases. In phase 1, we will conduct a survey-based study and anthropometric assessments (including 2D:4D ratio and BMI) in healthy young adults. Using validated questionnaires, we will collect self-reported data on sleep quality, sexual function, sleep chronotype, anxiety, and depressive symptoms. In phase 2, a subsample of phase 1 will undergo polysomnography and physiological and genetic assessments. Sleep architecture data will be obtained using portable polysomnography. The levels of testosterone, estradiol, progesterone, luteinizing hormone, follicle-stimulating hormone, prolactin, melatonin, and circadian regulatory proteins (circadian locomotor output cycles kaput [CLOCK], timeless [TIM], and period [PER]) and the expression levels of some miRNAs will be measured using blood samples. The rest and activity cycle will be monitored using actigraphy for a 7-day period.

**Results:**

In Poland, 720 participants were recruited for phase 1. Among these, 140 completed anthropometric measurements. In addition, 25 participants joined and completed phase 2 data collection. Recruitment from other sites will follow.

**Conclusions:**

Findings from our study may help to better understand the plausible role of PAE in sleep physiology, mental health, and sexual quality of life in young adults.

**International Registered Report Identifier (IRRID):**

DERR1-10.2196/29199

## Introduction

### The Activational and Organizational Hypothesis

Sexual differentiation of the brain largely occurs before birth, and this process primarily occurs during short periods when the brain is most sensitive to hormones. In humans, this happens in midpregnancy and the first 3 months after birth [1]. The *Organizational and Activational Hypothesis* suggests that the action of androgens during a sensitive period would have a permanent impact on the brain (*organizational* effect) and would eventually determine how one responds to gonadal hormones during and after puberty (*activational* effect) [2]. One well-documented evidence for this hypothesis is the sex difference in rodent sex behavior. Phoenix et al [3], in their seminal paper, reported that female rat pups that were exposed to testosterone during pregnancy displayed male sexual behavior in adulthood. Later, Feder and Whalen [4] found that male pups that were castrated shortly after birth (but still within the sensitive period) showed feminine sexual behavior as adults.

The exposure to androgens (eg, testosterone) before birth also influences the digit ratio (DR) or the ratio of finger lengths of the second and fourth digits (2D:4D) [5]. Studies on DRs were sparked by the study by Manning et al [6], which reported that the male DR is smaller than the female DR on average. The sex difference in DR appears in the first trimester and is thought to be influenced by androgens [7]. It remains relatively fixed during rapid growth in childhood and puberty. Therefore, postnatal DRs can be used as markers of prenatal androgen exposure (PAE).

Given the early event of sexual differentiation in the brain, various studies have shown that PAE, using DR as a marker, is associated with psychological conditions, including anxiety [8] and depression [9,10]. In this study, we plan to better understand the association of PAE with sleep and sexual functions as well as sexual attraction.

### Sleep and PAE

PAE may also influence sleep function in adults, but the evidence is minimal. Studies in rodents [11,12] indicated that, when treated with estradiol and progesterone in adulthood, male rats castrated neonatally (still within the sensitive period) have similar sleep outcomes (ie, nonrapid eye movement [nREM] sleep amount) to female rats, as compared with male rats orchiectomized as adults. This suggests that PAE may influence adult sleep function in response to sex steroid hormones. In addition, circumstantial evidence shows that lower PAE may be linked to more sleep difficulties, as insomnia is more common in females than in males [13]. Furthermore, if our DR hypothesis is supported by findings in this study (ie, androphilic attraction is associated with less PAE), our finding may help explain, from a biological perspective, why sleep deprivation is more common among men with androphilic attraction [14-16]. Undoubtedly, we also need to recognize that sleep problems in sexual minority populations can also be attributed to other factors such as discrimination and depression.

Verster et al [17] found no association between DR and sleep in females, but the right DR was positively correlated with total sleep time in males. However, the study had a number of limitations; for example, it did not include quantitative sleep measurements, and the analyses did not appear to take into account various factors such as age, BMI, and sexual orientation. In this study, we aim to conduct a rigorous study to determine whether PAE, indicated by the DR, is associated with subjective and objective sleep parameters.

### Sleep

Sleep is a state of unconsciousness, mandatory for everyone, that can be influenced by external auditory, sensory, or other stimuli. The role of sleep in general health has not been fully examined. Researchers connect sleep deprivation, especially chronic sleep loss, with numerous health problems such as obesity [18], diabetes [18,19], mental diseases [18,20], hypertension [21], cardiovascular events [22], and even common cold [23]. These relationships also point out some important roles of sleep in health. Sleep helps to maintain an efficient immune system [18,23,24], consolidate memory, and clean metabolites from the brain that aggregate during daytime [18]. Most importantly, it is necessary for life. Studies on both rats and Drosophila revealed that sleep loss lasting for too long ends up in death [18,25].

A healthy individual should spend approximately one-third of their day sleeping, stressing the importance of the process. The requisite sleep quality and quantity can change, but there is no specific boundary concerning sleep duration. This parameter varies both intra- and interindividually; thus, many factors determine the required amount of sleep. The American Academy of Sleep Medicine and Sleep Research Society, in their recent recommendation [26], achieved a consensus and established the minimum sleep duration for a healthy adult as 7 hours, although there may be individual variation. However, some studies [27,28] revealed that excessive sleep time might have detrimental health effects, such as increased cardiovascular mortality (concerning older adults). Short sleep duration can also have a negative health impact; for example, it increases the risk of hypertension [29]. Regarding this, sleep function may change because of aging. For example, many older adults commonly consider themselves to be less drowsy than young adults [30]. In contrast, sleep superficiality and fragmentation increase with age [31]. For many older adults, it takes longer to fall asleep, and the rapid eye movement (REM) phase is shorter, but the nREM phases 1 and 2 lengthen. Moreover, electroencephalogram (EEG) spectral power is lower in older adults [31].

Studies have shown that, besides age, sex is an important factor influencing the quantity of sleep [13,21,32]. The total sleep time among women is typically longer than that in men. Moreover, sleep efficiency (examined by polysomnography [PSG]) is higher in men than in women. Men are known to sleep lighter and get aroused more frequently. However, anecdotally, women report sleep disturbances more often [32].

### Sleep and Circadian Rhythms Assessment Methods

Sleep or circadian rhythms (CRs) can be examined using subjective and objective methods. The first method includes questionnaires. Examples of commonly used validated scales include the Pittsburgh Sleep Quality Index (PSQI) for assessing sleep quality measurement and the Morningness-Eveningness Questionnaire (MEQ) for measuring chronotype. Both are designed as self-assessment questionnaires [33-35]. PSQI distinguishes *good* and *poor* sleepers. It comprises 19 questions related to sleep quality, including latency, duration, or any sleep-related problems. The scale is divided into seven components. Each component can be rated from 0 to 3, and the total score ranges from 0 to 21. The higher the score, the more severe the sleep problem [33-35]. In contrast, MEQ is most frequently used for classifying morningness-eveningness types [36]. The MEQ categorizes patients into 5 groups, which differ depending on their chronotype [34]. The chronotype indicates an individual’s CR, which manifests itself as sleep-wake preferences. On the basis of 19 self-assessed questions, the scale distinguishes a definitely morning type, a moderately morning type, a neither or intermediate type, a moderately evening type, and a definitely evening type. Generally, morning types prefer to go to bed early and wake up early. However, if evening types wake up early, they would feel tired and are likely to get up with difficulty but are also more likely to fall asleep late in the night [37].

Actigraphy is another tool frequently used to objectively evaluate the sleep-wake parameters. The American Academy of Sleep Medicine recommends its use for examining people with sleep-wake disorders or sleep-disordered breathing [38]. An actigraph is a noninvasive wristwatch-like device that can measure rest-activity data from patients based on movements, and the data can then be analyzed by a software algorithm. However, this method could lead to an overestimation of sleep time and the number of awakenings [39]. In actigraphy, the sleep period is determined at the beginning of the immobility period, but it might not be the exact start of sleep. Nevertheless, actigraphy, if compared with PSG, is more convenient and shows a high accuracy (>80%) [40]; thus, it can be used as an alternative to PSG.

PSG, however, is commonly used as the gold standard for sleep examinations. If multiple channels are available, PSG can be coupled with EEG, electrooculogram, electromyogram, electrocardiogram, airflow, and oxygen saturation. Using PSG, every sleep phase could be distinguished and examined. An individual, when examined with EEG, electrooculogram, and electromyogram, presents three states: vigilance, nREM, and REM sleep [41-43]. One sleep cycle generally lasts between 90 and 110 minutes and is composed of a sequence of different sleep phases [44]. During nREM sleep, four stages are distinguished, in which the latter two stages are the deepest and are called slow-wave sleep. In one night, one could have multiple sleep cycles, but as the night progresses, shorter nREM episodes occur [45]. In one sleep cycle, the nREM phase lasts for 68-90 minutes (overall 75%-80% of night sleep), whereas REM sleep lasts for 5-30 minutes. Following a REM state, one will either turn into nREM state or wake up [46,47]. During the first cycle, REM sleep may last from 1 to 5 minutes; however, REM sleep episodes become progressively longer throughout the night [48].

### Neurochemical Modulation of Sleep

Sleep modulation and CR depend on a variety of factors. These concern both neuroanatomical structures (mainly suprachiasmatic nucleus [SCN]) and sleep-modulatory molecules (such as gamma amino butyric acid [GABA], adenosine, acetylcholine, and serotonin) [41] as well as external factors (eg, light and noise) [49]. All of the above participate in the two best-known mechanisms for controlling sleep, that is, a homeostatic and a circadian one. The first one is associated with increasing drowsiness after prolonged wakefulness, whereas the latter promotes wakefulness during the day and sleep at night. The circadian sleep process is based on the function of the SCN, located in the anterior hypothalamus, which receives information from the retina about light exposure and passes it to peripheral receptors via the neuroendocrine system, which will be described later [50]. Signaling in this region provokes vigilance, whereas loss of function causes fatigue and sleepiness [51].

SCN releases the neurotransmitter GABA, which promotes sleep [52,53]. However, the signal from SCN is not the only one needed to trigger nREM sleep. Falling asleep is also a consequence of the aforementioned homeostatic mechanism and adenosine build-up in the brain. Adenosine is a hypnogenic factor that progressively accumulates during wakefulness [42,43]. Moreover, some studies have suggested its importance in triggering nREM sleep by activating sleep-promoting neurons in the ventrolateral preoptic nucleus [43]. Neurons in the ventrolateral preoptic nucleus produce GABA and galanin, which inhibit arousal neurons in the hypothalamus and brainstem [52,54]. Switching from nREM to REM sleep is induced by acetylcholinergic neurons in the brainstem (located in the pedunculopontine tegmental and laterodorsal tegmental nuclei) as well as by GABA, serotonin, norepinephrine, and nitric oxide within this region [52].

Therefore, GABA plays a key role in sleep promotion. The level of GABA is increased during both nREM and REM sleep in comparison with waking values [53,55]. In contrast, acetylcholine concentration is higher in REM sleep than in nREM sleep and wakefulness [52]. In addition, various monoamines (eg, noradrenaline, dopamine, and serotonin) are also involved in sleep-wake cycle modulation. These molecules protect CR from unwanted changes. Not only the levels of molecules but also the expression of receptors that they bind to fluctuate within CR [49]. The activity of noradrenergic neurons in the locus coeruleus and serotoninergic neurons in the raphe nuclei decreases during nREM and even more during REM sleep [47].

### Endocrine Control of Sleep

The rhythmic secretion of most hormonal factors is governed by the internal biological clock and sleep state. Hormones can be divided into sleep-dependent hormones, such as growth hormone, prolactin, thyroid-stimulating hormone, and renin, or determined by CR-dependent hormones such as adrenocorticotropic hormone (ACTH), cortisol, and melatonin [56]. Interestingly, the SCN, which is called *the clock of the brain*, contains receptors for sex hormones [57].

In utero, gonadal embryogenesis occurs through certain genes (eg, *SRY*, *SF1*, *SOX1*, and *DAX1*). Between 10 and 20 weeks, in males, human chorionic gonadotropin is mainly responsible for testosterone production by Leydig cells [58]. This hormone leads to the differentiation of the Wolffian ducts to form the epididymis, vas deferens, seminal vesicles, and ejaculatory ducts. At approximately 8 weeks of pregnancy, the male fetus’s Sertoli cells start to produce Müllerian inhibiting substance, which leads to atrophy of the Müllerian ducts. The absence of Müllerian inhibiting substance and testosterone causes sexual differentiation in women. Female sexual differentiation appears to occur without hormonal stimulation [59].

In adults, the plasma levels of various hormones vary according to the sleep stage or CR [56]. The hypothalamic-pituitary-adrenal axis controls the production and secretion of ACTH. The hypothalamus produces a corticotropin-releasing hormone, which stimulates the anterior pituitary to release ACTH. ACTH acts on the adrenal cortex and affects cortisol and adrenal androgen levels. The increase in cortisol provides a negative feedback loop to the hypothalamus, and consequently, the level of corticotropin-releasing hormone decreases [60]. Sleep onset is associated with decreased cortisol secretion. With increasing hours of sleep, there is an elevation of cortisol levels, which subsequently decreases throughout the day after awakening [56].

The gonadotrophin-releasing hormone is synthesized in neurons within the hypothalamus. Its main function is to stimulate the anterior pituitary gland to release follicle-stimulating hormone (FSH) and luteinizing hormone (LH) [61]. In females, the menstrual cycle is divided into follicular and luteal phases. The former begins on the first day of menstruation. At the beginning of this phase, the ovary secretes a small amount of hormones that leads to low estradiol and progesterone levels. In the midfollicular phase, FSH starts to increase and stimulates the secretion of estradiol. Eventually, the increase in estradiol levels leads to a negative feedback loop. Estradiol peaks one day before ovulation. At this time, a unique endocrine phenomenon occurs as a switch from negative feedback to positive feedback, resulting in a midcycle surge. There is an increase in LH, as well as FSH, but in the case of FSH, the increase is not as high. Progesterone is then released by the corpus luteum. This hormone is responsible for increasing the temperature and relaxing the uterine smooth muscle. Later in the luteal phase, if the oocyte is not fertilized, LH, progesterone, and estradiol levels will decrease. However, estradiol is important for developing secondary sex characteristics in women. It has been suggested that low estrogen level has serious implications such as cardiovascular responsiveness, insulin resistance, and obesity [61].

In males, LH binds to receptors on Leydig cells and stimulates them to produce testosterone. In contrast, FSH acts on Sertoli cells to help promote spermatogenesis. Testosterone can be further converted into two forms: estradiol or 5α-dihydrotestosterone. Its production is implicated in the development of the male phenotype in embryos, providing sexual maturity at puberty and sexual function. In addition, both testosterone and estradiol inhibit the release of gonadotrophin-releasing hormone by the process of negative feedback [61]. In summary, in males, LH is responsible for testosterone production by the testes, and FSH stimulation is important for normal spermatogenesis.

In females, LH and FSH stimulate ovarian production of estradiol and progesterone, which have an impact on sleep changes during the menstrual cycle. There is a pulsatile rise in their levels at sleep onset in both sexes. During prepuberty and puberty, their levels increase during sleep. In addition, hormonal fluctuations during the menstrual cycle have an impact on sleep, for example, by decreasing the REM stage in the luteal phase and increasing sleep stage 2 in the midluteal phase [56]. There is a significant decrease in sleep efficiency and sleep quality as well as an increase in sleep onset latency during the luteal phase.

The association between reproductive hormones and sleep has been studied previously; however, further research is needed. One evidence comes from the fact that hormonal profiles vary between sexes, and there are several sex differences in sleep patterns. Women generally need more sleep, spend more time in bed, and sleep longer. They also report more sleep difficulties, but by actigraphy, women have better sleep quality compared with men [56]. Furthermore, recent studies have shown that sex hormones have different impacts on CR and sleep physiology in males and females. In female rats, estradiol promotes arousal in the active phase of sleep, but after sleep loss, both estradiol and progesterone selectively facilitate REM rebound while reducing nREM intensity [62]. A similar effect of estradiol in facilitating REM rebound has also been shown in castrated male rats [63]. However, testosterone influences the organization of CR and the timing of sleep. For example, in young men, higher baseline testosterone levels are associated with the evening chronotype [64]. It is also believed that testosterone concentration decreases during the day and peaks after 90 minutes of sleep in the evening, coinciding with the first REM phase of sleep [65]. In a cross-sectional analysis, no association was found between serum levels of various hormones (free testosterone, bioavailable testosterone, total testosterone, and sex hormone–binding globulin) and sleep quality [66]. However, impaired sleep and elevated BMI were associated with low testosterone levels in a study of 9756 men aged 16-80 years [67].

Several studies have explored the relationship between sleep and reproductive hormone secretion [68-70]. In one such study, a significant association was found between FSH levels and sleep duration in women with normal cycles. FSH levels were 20% higher in long-time sleepers than in short-time sleepers [71].

### Genetic Basis of Sleep and CRs

Various genes may also influence sleep and CR. For example, CR relies on the functioning of the *CLOCK:BMAL1 (*brain and muscle ARNT-Like 1*)* complex and the expression of genes such as CLOCK (*circadian locomotor output cycles kaput*), *PER* (*period*), *CRY* (*cryptochrome*), and *TIM* (*timeless*). The *CLOCK:BMAL1* complex is formed by two positive regulators. They bind to the DNA during the day to promote *PER* and *CRY* expression. In the evening, PER combines with CRY in the cytoplasm, then migrates to the nucleus and subsequently inhibits *CLOCK:BMAL1* and, in turn, its own transcription. Afterward, the heterodimer PER/CRY disintegrates; thus, *CLOCK:BMAL1* is not repressed. This cycle is called a transcription/translation feedback loop, which is repeated every 24 hours [50,72,73]. Of note, the expression of the PER/CRY complex is not the only process regulated by *CLOCK:BMAL1* [50,73]; however, it is the core one that modulates CR [50].

The exact roles of the aforementioned gene products are still not well defined. However, studies have shown that PER plays an important role in maintaining appropriate circadian timing [50]. *PER3* gene polymorphism affects sleep homeostasis, and pathological loss of *the PER1 and PER2* genes in mice leads to increased sleep pressure [74]. Furthermore, the four-repeat *PER3* allele, compared with the five-repeat allele, is associated with decreased slow-wave sleep and better cognitive performance after sleep deprivation [75]. In addition, the double knockout of *CRY1-2* in mice results in an increased nREM sleep phase and complete dysregulation of CR. Moreover, an insufficient *CLOCK* gene disrupts CR in mice under constant darkness [74]. However, full knockdown of *TIM* causes death in mammalian fetuses, and conditional knockdown promotes circadian arrhythmicity in the SCN [76,77]. Mutations in any of the *PER*, *CRY*, or *TIM* genes are considered to cause familial advanced sleep phase syndrome, in which patients wake up and go to bed early, therefore presenting features of morning chronotype [78].

In peripheral blood, products of circadian genes expression can be found at a concentration similar to that of SCN (obtained from mononuclear cells).

### miRNAs and Circadian Clock

Epigenetics provides new insights into the circadian clock. The commonly known types of epigenetic changes involve the expression of noncoding RNAs, such as circulating miRNAs [79,80]. The following miRNAs may have an important role in altering CR: miR‐132, miR‐219, miR‐192, miR‐194, and miR-34a [81-83]. Studies of rodents have shown that levels of miR‐132 are associated with light‐dependent resetting of the circadian clock, and miR‐219 was shown to maintain the length of the circadian period [81]. In addition, miR‐192 and miR‐194 (miR‐192/194) are powerful regulators of PER family members (*PER1*, *PER2*, and *PER3*) [83]. The overexpression of these miRNAs may inhibit the synthesis of PER proteins, resulting in an altered CR [83]. In addition, overexpression of miR-34a is associated with a decrease in the synthesis of PER1 protein [82].

### Body Composition and Sleep Quality

Due to the global obesity problem, an understanding of the factors significantly related to body weight and composition is needed. Obesity is currently one of the most common causes of health problems in developed countries, including Poland, New Zealand, Japan, and the United Kingdom [84]. According to the latest results of the European Health Interview Survey, people in Poland who are overweight and obese constitute 37.5% and 17.2% of the total population aged ≥18 years, respectively. These results were above the average for the 28 European Union countries, which is 35.7% of people who are overweight and 15% of people with obesity [85].

A variety of factors, such as physical activity, exposure to stress, and eating habits as well as sleep quality, may affect body composition. Sleep is an essential element in the regeneration of the body and provides appropriate management of energy resources [86]. Short sleep leads to more frequent use of stimulants, which is also associated with the deposition of more fatty acids [87]. People with poor quality of sleep are more likely to have more body fat and a predisposition to obesity [88]. In addition, people who sleep less also tend to accumulate adipose tissue [89]. The DR (2D:4D) is commonly linked with body components, especially muscle body content [90] and fat tissue [91]. Higher PAE, as indicated by a lower DR, is associated with a higher content of muscle tissue among adult men [92] and children [93]. In addition, a higher DR (indicative of lower PAE) is associated with excess body fat, independent of the stage of life [93,94].

### Sleep and Sexual Function

Another goal of this study is to explore the association between sleep quality and sexual function. Sleep problems are common in many populations [95,96], which may reflect daily lifestyles. People may experience acute sleep loss in a single night, but chronic sleep deprivation, where people have inadequate sleep over several consecutive days, can also occur. Chronic sleep deprivation can detrimentally affect health by increasing the wear and tear of various body systems (ie, the allostatic load) from repeated consecutive sleep loss [97]. This cumulative stress can negatively affect metabolic, immune, and psychological health [97,98].

Sexual inactivity is also becoming common in some societies [99,100]; however, few studies have explored the relationship between sleep quality and sexual function. Given that the main brain center for male sexual behavior (ie, the preoptic area [101]) is also a sleep-promoting area [102,103], there is a possible biological link between these two functions.

Several preclinical studies have explored the impact of sleep deprivation on male sexual behavior. However, these studies mainly used the same sleep deprivation paradigm (ie, 96 hours of REM sleep deprivation). The findings from these studies were inconsistent, including no effect [104,105], impairment [106,107], and even improvement [108] of male sexual behavior. Interestingly, the earliest study on the effect of REM sleep deprivation on male sexual behavior found that rats that were *low* copulators exhibited an increase in their sexual activity following a week of REM sleep deprivation [109]. Unfortunately, the data from that study were only published as an abstract, so the experimental details were not reported. Recently, one of the authors (EW) found that daily mating sessions for 2 weeks dampened sexual behavior in male rats. However, the muted sexual response lasted longer in rats that were chronically sleep deprived (kept awake for 4 hours per day for 1 week) [110].

Human studies have also documented an association between sleep deprivation and impaired sexual function. For example, Seehuus and Pigeon [111] found an association between insomnia symptoms with lower intercourse satisfaction in males as well as with sexual arousal and vaginal lubrication in females. In addition, Kalejaiye et al [112] showed that men with obstructive sleep apnea reported worse erectile function than those without the condition. Furthermore, a recent study found bidirectional associations between insomnia symptoms and orgasm difficulty [113]. The analyses in that study were controlled for age, number of comorbidities, BMI, past use of androgen deprivation therapy, daytime sleepiness, fatigue, and depressive and anxiety symptoms. Furthermore, obstructive sleep apnea has been associated with erectile dysfunction in men, and the use of continuous positive airway pressure, which improves sleep quality, may improve erectile function [114].

Currently, however, there is little information on how objective sleep parameters (eg, sleep stages, sleep or wake latencies, and number of awakenings) are linked to sexual function in healthy populations. However, determining this would have clinical relevance as it may help indicate which sleep-related outcomes need to be improved to increase sexual function.

In this study, we will conduct both subjective and objective assessments of sleep as well as collect sexual function data using validated questionnaires, with the aim of finding associations between sleep and sexual parameters. Subjective sleep quality will be measured using the PSQI [33]. Objective sleep measurement will be performed using a portable PSG system (Nox 1A, ResMed), which can monitor various sleep parameters, including total sleep time and duration of REM and nREM phases. In addition, we will include an MEQ [115] to help indicate participants’ chronotype (ie, whether one is more of a *morning* or *evening* person), as people with sleep problems are less likely to be a *morning* person [116-119].

Sexual function will be assessed using the Arizona Sexual Experience Scale, which is a brief five-item scale measuring self-reported information on the strength of sex drive, how easy it is to be sexually aroused, to get and maintain an erection, to reach an orgasm, and orgasm satisfaction [120].

### DR and Sexuality

Some data support the Organizational and Activational Hypothesis on human sexuality, but these studies have limitations. For example, the majority (93%) of 46XY individuals with androgen insensitivity syndrome are androphilic, that is, attracted to men [121]. Additional evidence comes from women with congenital adrenal hyperplasia who have higher levels of androgens than normal and a higher likelihood for same-sex attraction than women without the condition [122]. This suggests that androgens play a role in gynephilia, that is, attraction to women. However, these data should be considered with caution. For example, the study by Wisniewski et al [121] had a small sample size of 14, and in the study by Meyer-Bahlburg et al [122], not all women with congenital adrenal hyperplasia had a sexual attraction toward women.

Many studies have explored the association between DR and sexual orientation. Breedlove and his team [123] were the first to conduct such a study, where it was found that the DR of the right hand in gynephilic women was smaller than that of androphilic women, but the study reported no such difference between androphilic and gynephilic men. Since then, several studies have explored similar associations in both sexes, and the review by Grimbos et al [124] found that androphilic women, on average, have larger (ie, more female typical) DRs than gynephilic women. This finding suggests that the attraction to females in gynephilic women may be mediated, at least partially, by exposure to elevated levels of prenatal androgens. However, the data for males are less consistent [125-130]. It is still worth noting, though, that some studies have found that heterosexual men have a smaller DR on average than homosexual men [125-127,130]. Again, this may suggest that the androphilic attraction in males may be attributed, at least partially, to lower PAE.

Overall, studies on the relationship between DR and sexual orientation remain controversial [123,131,132]. This is not surprising, as biological studies on sexual orientation could potentially be political [133], with some people of the view that sexual orientation is predominantly socially determined rather than biologically based. We recognize that all theories on the biological basis of sexual orientation have caveats. However, a controversial topic should not stop researchers from further investigations in this area. Indeed, further studies are crucial to provide evidence, be it biological or social, in an objective way.

Several factors have been discussed regarding the limitations of assessing DR. One aspect, which is often noted, is the fact that the effect size is not large, and there is no cut-off indicating male or female pattern of DR; thus, there is a large overlap in the distribution of DR between sexes. This is also true for studies exploring differences in DRs of people with various sexual orientations. In addition, many studies that attempted to find an association between DR and sexual orientation have collected DR data through indirect methods, such as from photocopies or scans. However, it is now known that there is a potential discrepancy between direct and indirect measurements of DR [134]. Furthermore, these studies explored the relationship between sexual orientation and biological but nonbiological factors, such as social factors, which some researchers have suggested to play a role in sexual orientation [133], were not accounted in those studies.

In addition, studies on sexual orientation and DR have rarely considered two important factors: (1) handedness and (2) sexual attraction (how attracted one is to the same and opposite sex) *per se* rather than sexual identity (eg, gay, bisexual, and heterosexual). It is important to consider handedness, as nonheterosexual populations are more likely to be non–right-handed [135]. However, studies often only analyze data based on the left or right hand. Regarding sexual attraction, studies have often analyzed data based on sexual identity (eg, heterosexual, homosexual, and bisexual). These categories can be subjective. Considering that DR is a continuous variable, researchers need to consider DR data based on the degree of sexual attraction (ie, how much attraction one has toward the opposite sex and the same sex). The Kinsey Scale, which was originally used to determine the degree of sexual activity with the opposite and same sex, could potentially be adapted for a degree of sexual attraction. In this study, we aim to better understand the association between PAE (indicated by DR) and sexual attraction (not sexual identity) by taking into account the participants’ handedness information.

Finally, we are not aware of any previous or pending studies that have associated DR with sexual function *per se* (eg, sexual desire, erectile function, and orgasm). Therefore, we will investigate this topic as well. This will help indicate the extent to which PAE plays a role in determining sexual function in adulthood.

### Objectives

#### Objective 1

We will explore the association between PAE as indicated by the DR and sleep function in young adults. This research will have two phases to address this objective. In phase 1, sleep parameters will be assessed using a validated questionnaire (subjective measure). In phase 2, we will determine if PAE is related to objective sleep measures (using actigraphy and portable PSG), along with sleep-related correlates such as hormones, circadian regulatory proteins, and body composition.

#### Objective 2

Here, we will explore if sleep quality is associated with sexual functions. We will collect both objective and subjective sleep parameters. Sexual function data will be assessed using validated questionnaires. In addition, as noted above, we will investigate the extent of how PAE is associated with sexual function in young adults.

#### Objective 3

Finally, we will determine the association between PAE (indicated by DR) and sexual attraction, while taking into account the participants’ handedness information. Our finding may help show whether handedness information (which is associated with hormones and brain lateralization) can help explain the inconsistencies in past findings of DR and sexual orientation studies.

## Methods

### Recruitment

This research will be undertaken in two phases, with parallel recruitments in 4 different countries. Five institutions (Medical University of Lodz, Poland; University of Lodz, Poland; University of Otago, New Zealand; Aichi Medical University, Japan; and Swansea University, United Kingdom) are participating in this study.

### Phase 1

**Figure 1:**
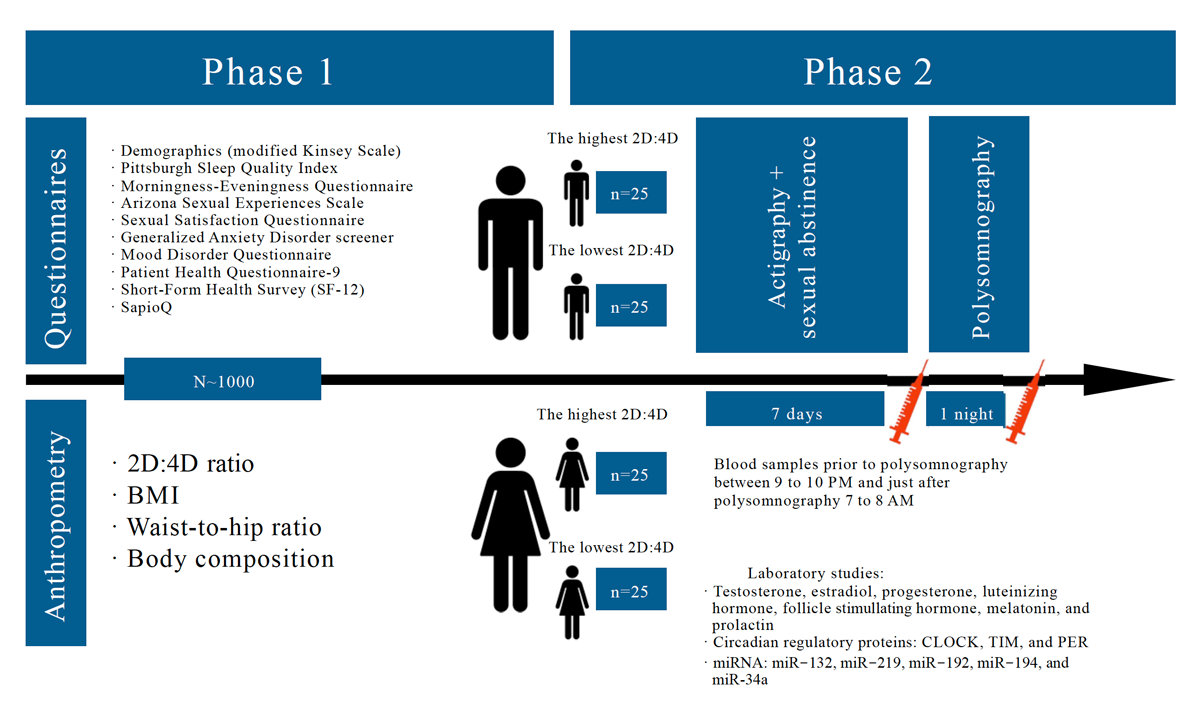
Flowchart of the study. CLOCK: circadian locomotor output cycles kaput; PER: period; TIM: timeless; 2D:4D ratio: ratio of the second digit length to the fourth digit length.

As shown on Figure 1, in phase 1, we plan to recruit at least 1000 participants from each site. Eligibility criteria are listed in Textbox 1. The large sample is required because of the third aim, where we intend to recruit participants from sexual minorities (ie, gay, bisexual, and lesbian), which can be challenging. In one study [22], about 150 participants per group were sufficient to detect a significant difference of approximately 10% in the DR of heterosexual and nonheterosexual people. In a recent survey at the University of Otago [136], approximately 28% (356/1234) of students were identified as sexual minority. If 30% of our participants are sexual minority, that would be approximately 300 participants (150 male and 150 female) out of 1000.

**Inclusion and exclusion criteria.**

**Inclusion Criteria**

- A full understanding of the study rules by the participant was confirmed by written informed consent to participate in the study
- Age range: 18-30 years

**Exclusion Criteria**

- Diagnosis of chronic, hormonal, and mental health conditions
- Pregnancy and lactation
- Taking long-term medicines (including hormonal contraception)
- Injuries to the fingers of the upper limbs
- Deformation of the fingers of the upper limbs
- Diseases leading to deformation of the fingers of the upper limbs
- Lack of consent or inability to follow recommendations related to participation in the study

Upon consenting, participants will need to do the following:

1. Complete questionnaires on demographics (eg, age, ethnicity, sex, gender, sexual attraction using the Kinsey Scale, sexual orientation, and handedness); PSQI; MEQ; Arizona Sexual Experience Scale; Sexual Satisfaction Scale; Generalized Anxiety Disorder screener; Mood Disorder Questionnaire; Patient Health Questionnaire-9, short-form health survey; and SapioQ.
2. Have anthropometric measurements (body weight, height, BMI, finger length: index and ring fingers, waist, hips, and neck circumference) recorded.
3. Body composition analysis (InBody 270) will be used to determine fat and muscle mass. We include this analysis because obesity is linked to sleep-related breathing disorders as well as insomnia. Noninvasive, easy, and common body impedance analysis method is based on measuring the electrical impedance in various body tissues, that is, the sum of geometric resistance (active resistance) and reactance (passive resistance). Bioelectrical impedance analysis is used to assess the values of the body components, such as fat-free mass (%), fat mass (%), muscle mass (%), and total body water (%). In addition, segmental lean and fat distribution can be measured to assess tissue distribution in each quarter of the body. The basic metabolic rate is calculated. Before each measurement, foot, feet, and hands must be wiped using wet wipes to increase electrical conductivity. During the measurement, the individual should be in an upright position [137].

### Phase 2

#### Overview

When phase 1 is completed, we will follow up with those interested in phase 2. For this phase, we estimate that we will need at least 50 male and 50 female participants, regardless of sexual orientation. Using reference data from the PSQI, we estimate that, for each sex, we will need 25 participants with DR<1.0 (indicative of high PAE) and 25 participants with DR>1.0 (indicative of low PAE) to detect a 50% difference in sleep quality, with an α level of .05 and a power of 0.8.

Once consented, each participant will need to do the following:

1. A 1-week quantitative daily and nocturnal activity recording using portable actigraphy. On the basis of the actigraphic record, it will be possible to evaluate parameters such as the average level of activity during the day and at night, the amount of time spent actively and inactively during the day and night, average sleep time, sleep continuity, number of awakenings during sleep, and number of naps during the day. On the last day, one-night PSG data (total sleep time, duration of REM and nREM phases, apnea-hypopnea index, arousal index, and saturation) will be captured with a Nox A1 (ResMed). During 1-week actigraphy, all participants will have to agree to sexual abstinence because of blood samples before and just after PSG.
2. Blood collection, before and after the polysomnographic night for assessment of various hormones (testosterone, estrogen, estradiol, progesterone, LH, FSH, and prolactin) and circadian regulatory proteins (eg, CLOCK, PER, and TIM) as well as selected miRNAs.

#### Enzyme-Linked Immunosorbent Assay

The levels of the investigated proteins will be assessed using enzyme-linked immunosorbent assay kits. Each assay will include six standard concentrations, and every sample will be run in duplicate. The concentration will be assessed using a microplate reader to measure the absorbance at 450 nm. The standard curves will be created using the four-parameter logistic method. All samples should be within the assay range in accordance with the information supplied by the kit manufacturer. An interassay coefficient of variation of less than 15% is acceptable. The intra-assay coefficient of variation values were less than 10%.

#### miRNA

The common miRNA procedure includes the following steps: RNA isolation, reverse transcription, and quantitative polymerase chain reaction using appropriate starters for each miRNA. An RNA/miRNA purification kit is needed to isolate miRNAs. A reverse transcription kit was used to perform reverse transcription. The analyses will be performed according to the protocol supplied by the manufacturer, using an appropriate amount of RNA in each sample. The reactions will be performed in a thermocycler according to the reaction parameters supplied by the manufacturer. Furthermore, we will dilute the obtained cDNA according to a protocol using RNAse-free and DNAse-free water. The final step will be quantitative polymerase chain reaction analysis for each sample in duplicate and each miRNA (using proper starters) using a real-time polymerase chain reaction machine. The obtained results will be analyzed using the software that calculated the cycle threshold (Ct) for each sample. The samples over Ct=39 cycles will be excluded from further analyses. The spike in the kit is used to normalize the expression levels of the obtained miRNAs. The delta cycle Ct method will be used to make a final calculation using the following formula: 2(mean CtmiR − mean Ct of reference) [138].

### Data Analyses

Demographic data will be summarized with descriptive statistics.

#### Objective 1

Subjective sleep measures and sexual data from phase 1 as well as objective sleep measures and its (hormonal, molecular, and psychological) correlates from phase 2 will be compared between participants with DRs<1.0 (indicative of high PAE) and participants with DR>1.0 (indicative of low PAE) for each biological sex, while taking into account their handedness.

#### Objective 2

Multiple regression analyses will be performed to determine the relationship between sleep and sexual parameters while controlling for age, BMI, psychological health (anxiety and depression), chronotype, and sexual orientation.

#### Objective 3

For each sex, DR data will be categorized by handedness and correlated with sexual attraction (measured using the Kinsey Scale).

## Results

In 2020, we started the first and second stages of our study in Poland. In phase 1, we recruited 720 participants, of whom 140 underwent anthropometric measurements. The second stage started in February 2021, and since then, 25 participants have completed the data collection for phase 2. We expect to complete the data collection for phase 2 in 2022.

## Discussion

Data from this study will provide some information on how PAE is associated with sleep and sexual functions as well as sexual attraction. Many factors may contribute to sleep and sexual problems. However, our research should provide further insight from a biological perspective on how early-life hormonal factors can have a long-term impact on these functions. Furthermore, we will obtain information on the role of PAE in sexual attraction.

## Appendix

Multimedia Appendix 1 Peer-reviewer report from the National Science Centre, Poland.

## Abbreviations

ACTH: adrenocorticotropic hormone
BMAL1: brain and muscle ARNT-Like 1
CLOCK: circadian locomotor output cycles kaput
CR: circadian rhythm
Ct: cycle threshold
DR: digit ratio
EEG: electroencephalogram
FSH: follicle-stimulating hormone
GABA: gamma amino butyric acid
LH: luteinizing hormone
MEQ: Morningness-Eveningness Questionnaire
nREM: nonrapid eye movement
PAE: prenatal androgen exposure
PER: period
PSG: polysomnography
PSQI: Pittsburgh Sleep Quality Index
REM: rapid eye movement
SCN: suprachiasmatic nucleus
TIM: timeless
