# Supplementary material for: Understanding the Associations of Prenatal Androgen Exposure on Sleep Physiology, Circadian Proteins, Anthropometric Parameters, Hormonal Factors, Quality of Life, and Sex Among Healthy Young Adults: Protocol for an International, Multicenter Study
Source: JMIR Res Protoc. 2021 Oct 6;10(10):e29199. doi: 10.2196/29199 (PMC8529469; doi:10.2196/29199)
Supplement: Multimedia Appendix 1 [file resprot_v10i10e29199_app1.pdf]

Physiology of sleep, miRNA, circadian proteins, and the assessment of anthropometric and hormonal factors, quality of life, and sex among young healthy adults – BOAT international, multicentre study protocol.

ELIGIBLE APPLICATION FOR FINANCING
The application has been qualified for financing. Congratulations! We would like to remind you that the basis for the settlement of the project is the performance of a scientific activity, in accordance with the general terms of the contract.

Please be advised that in accordance with § 12, chap. 4 of the Terms and Conditions of awarding funds for the implementation of tasks financed by the National Science Center in the MINIATURE 4 call for a single scientific activity, a person carrying out a scientific activity under this application may not apply for re-funding under the MINIATURE competition.

SUBSTANTIVE EVALUATION OF THE APPLICATION
Does the research carried out by the person to carry out the scientific activity meet the criterion of basic research? **YES**
Does the research activity meet the other requirements set out in the call notice? **YES**
Are the planned costs of the scientific activity justified? **YES**

A. ASSESSMENT OF THE ACCOUNT OF THE PERSON INTENDED TO IMPLEMENT THE SCIENTIFIC ACTIVITY 20%
Assessment of the achievements of the person intended for the implementation of the scientific activity (1 - 5)

Reviewer 1:
2 Average
Dr. Wojciech Kuczyński presented a list of detailed information about the course of his professional and scientific career, including obtaining a doctoral degree in 2019. The research paper selected for evaluation was published in the Scientific Reports journal, belonging to Q1 in the JCR multidisciplinary research category. The publication, in which the applicant is not the lead author or the corresponding author, relates to the results of a descriptive case-control study that assessed the relationship between the 2D: 4D finger length index and the incidence of lung cancer.

Reviewer 2:
4 Very good
The author of the application defended his doctoral dissertation with distinction in 2019. His scientific achievements include 8 publications and 14 congress reports in the country and abroad. He is the co-author of the clinical trial "SOREST 2019". As part of the Erasmus program, he spent 18 months at two Italian universities, and also completed two research internships in Japan. He was the author of the UMED grant for young scientists.

Reviewer 3:
4 Very good
The author of the project is a young, ambitious scientist with already very good publishing achievements. He has had research internships and research collaborations abroad. The application shows a very good scientific publication. The principal investigator is neither the first nor the last author, but it can be assumed that he made a significant contribution to collaborative research.

B. ASSESSMENT OF THE SCIENTIFIC ACTIVITY 80%
Merit-based evaluation of the scientific activity (1 - 5)

Reviewer 1:
2 Average
The research project concerns a pilot study aimed at assessing the correlation between selected clinical, laboratory, anthropometric parameters and sleep physiology. The way of describing the target, very comprehensive study for which the applicant will collect preliminary results on the basis of the Miniature project is very complex and unstructured. The preliminary research did not present the reasons for choosing the size of the studied population. It is also difficult to see what reasons were

behind the selection of specific laboratory determinations for the implementation of this project and the omission of others (e.g. melatonin). There is also no justification as to why the reagents for the assays of selected miRNAs, planned for the purposes of the study, were not included in the laboratory materials. In the case of ELISA determinations, the discrepancy between the size of the kit (96 samples) and the size of the population (n = 100) was not specified.
Reviewer 2:
5 Distinguishing
The aim of the presented application is to describe the relationship between hormonal indices, circadian rhythm regulatory proteins and anthropometric indices, body composition, mental health and sexual intercourse of young adults and the structure of sleep, its latency and chronotype. The choice of the analyzed indicators is well justified.

Reviewer 3:
4 Very good
As part of the project, the author planned an assessment of the relationship between biochemical parameters, circadian rhythm regulatory proteins, body composition and mental health, sexual life and sleep structure and chronotype. The research will be carried out with both laboratory tests and questionnaires. The planned experiments will allow the collection of a large amount of data and, based on multivariate analysis, will certainly provide new information about sleep physiology, which will be an excellent basis for applications for further projects.

C. RECOMMENDATION
Should the application go for funding?

Reviewer 1:
0 I do not recommend financing

Reviewer 2:
2 I recommend for financing
The amounts listed in the cost estimate are reasonable. The author of the project - His achievements so far seem to guarantee the implementation of the project. The designed research is important and valuable, as it touches upon the basic issues related to sleep, the disturbances of which constitute a significant medical problem of underestimated importance.

Reviewer 3:
2 I recommend for financing

SUMMARY OF THE EVALUATION OF THE APPLICATION
Reviewer 1
Summary of the application assessment
The aim of the research application is to obtain the preliminary results necessary for the proper planning of the future multicentre study. Unfortunately, the presented methodology and the course of the research raise doubts as to the possibility of obtaining actual results useful for such a purpose.

Reviewer 2
Summary of the application assessment
The presented research project is part of a multi-center research program involving 5 universities - UMED, and universities from Great Britain, New Zealand and Japan. The author of the project is its manager. This is an additional guarantee of a favorable implementation of the planned research. They concern the study of various dependencies related to sleep. Taking into account the prevalence of disorders of this basic mechanism of regeneration of the organism, I consider it most advisable and advisable to carry out the research.

Reviewer 3
Summary of the application assessment
Very good conclusion, the project manager is a young and ambitious scientist with good achievements. The project cost estimate is well estimated and reasonable. The manager's experience indicates the correct implementation of research and the settlement of the project with publications.
